# Giant ulnar artery aneurysm secondary to congenital arteriovenous fistula and trauma: a case report

**DOI:** 10.1093/jscr/rjag685

**Published:** 2026-08-11

**Authors:** Yusuke Hagiwara, Masayoshi Otsu, Takuto Maruyama, Shintaro Abe, Katsuyuki Yoshida, Hiroyuki Watanabe

**Affiliations:** Department of Cardiovascular Surgery, Japanese Red Cross Narita Hospital, Chiba, Japan; Department of Cardiovascular Surgery, Japanese Red Cross Narita Hospital, Chiba, Japan; Department of Cardiovascular Surgery, Japanese Red Cross Narita Hospital, Chiba, Japan; Department of Cardiovascular Surgery, Japanese Red Cross Narita Hospital, Chiba, Japan; Department of Cardiovascular Surgery, Japanese Red Cross Narita Hospital, Chiba, Japan; Department of Cardiovascular Surgery, Japanese Red Cross Narita Hospital, Chiba, Japan

**Keywords:** ulnar artery aneurysm, arteriovenous fistula, endoaneurysmorrhaphy, arteriogenesis, ultrasound nerve mapping

## Abstract

An 80-year-old woman presented with a giant ulnar artery aneurysm that had developed secondary to a congenital arteriovenous fistula and trauma. The aneurysm progressively enlarged over 33 years, reaching 68 × 88 mm with a forearm circumference of 31 cm. Despite proximal ulnar artery ligation performed three years prior, the aneurysm continued to enlarge due to collateral circulation arising from a tortuous segment of the brachial artery. Definitive surgical intervention was undertaken, comprising collateral vessel ligation, brachial artery reconstruction, and endoaneurysmorrhaphy with removal of 235 g of organized thrombus. Histopathological examination revealed transmural sclerosis consistent with an atherosclerotic aneurysm. Preoperative ultrasound mapping of neurovascular structures facilitated safe dissection in the setting of distorted anatomy. Postoperatively, forearm circumference improved to 25 cm. Residual aneurysm perfusion was identified on contrast-enhanced computed tomography, highlighting the technical complexity of achieving complete exclusion in giant aneurysms with multiple inflow sources.

## Introduction

Ulnar artery aneurysms are relatively uncommon. Trauma is the most frequent etiology, as exemplified by hypothenar hammer syndrome. Other reported causes include atherosclerosis, vasculitis, and infection. Although the indications for surgical intervention remain controversial, surgery is generally recommended for symptomatic patients or those at risk of embolism [1]. Congenital arteriovenous fistula (AVF) is an exceptionally rare cause of peripheral arterial aneurysm, with only isolated cases of congenital AVF-associated ulnar artery aneurysm reported in the literature [2].

We report a case of an ulnar artery aneurysm that developed secondary to a congenital AVF and trauma. The aneurysm progressively enlarged and became symptomatic over a 33-year period, necessitating surgical intervention.

## Case report

**Figure 1:**
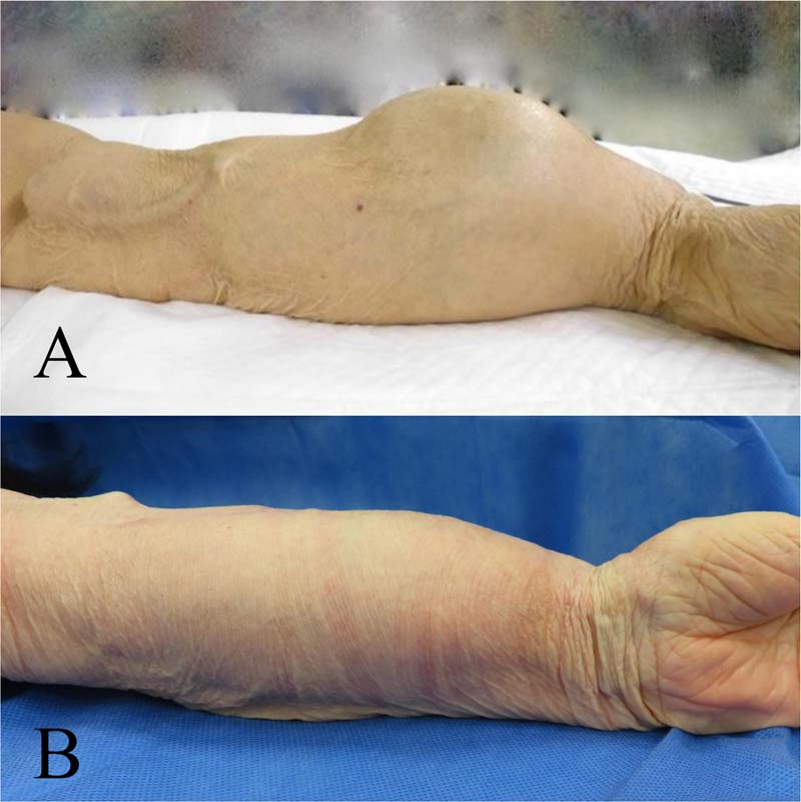
Preoperative and postoperative appearance of the left forearm. (A) Preoperative photograph demonstrating marked swelling of the left forearm due to the giant ulnar artery aneurysm. (B) Photograph taken 8 months postoperatively, showing significant reduction in forearm swelling following surgical intervention.

An 80-year-old woman had been diagnosed with a congenital AVF 43 years prior. She sustained a distal radius fracture of the left wrist in a bicycle accident 33 years prior, after which swelling of the left forearm gradually worsened. She was diagnosed with an ulnar artery aneurysm but remained asymptomatic and was managed conservatively. Over 33 years, the aneurysm enlarged to 64 × 76 mm, and the forearm circumference reached 29 cm (Fig. 1).

**Figure 2:**
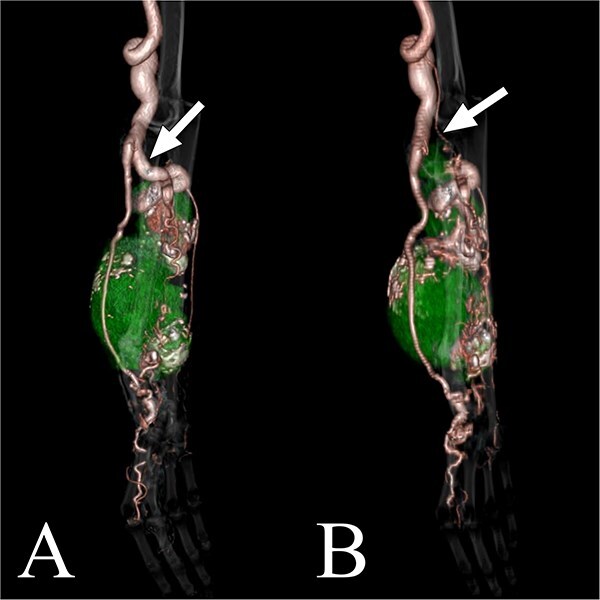
Three-dimensional CT angiography before and after proximal ulnar artery ligation. (A) Preoperative imaging demonstrating the ulnar artery aneurysm. The arrow indicates the ulnar artery at the site of ligation. (B) Imaging obtained 7 months postoperatively. Despite proximal ligation, the aneurysm persisted, and the arrow indicates a newly developed collateral vessel supplying the aneurysm.

**Figure 3:**
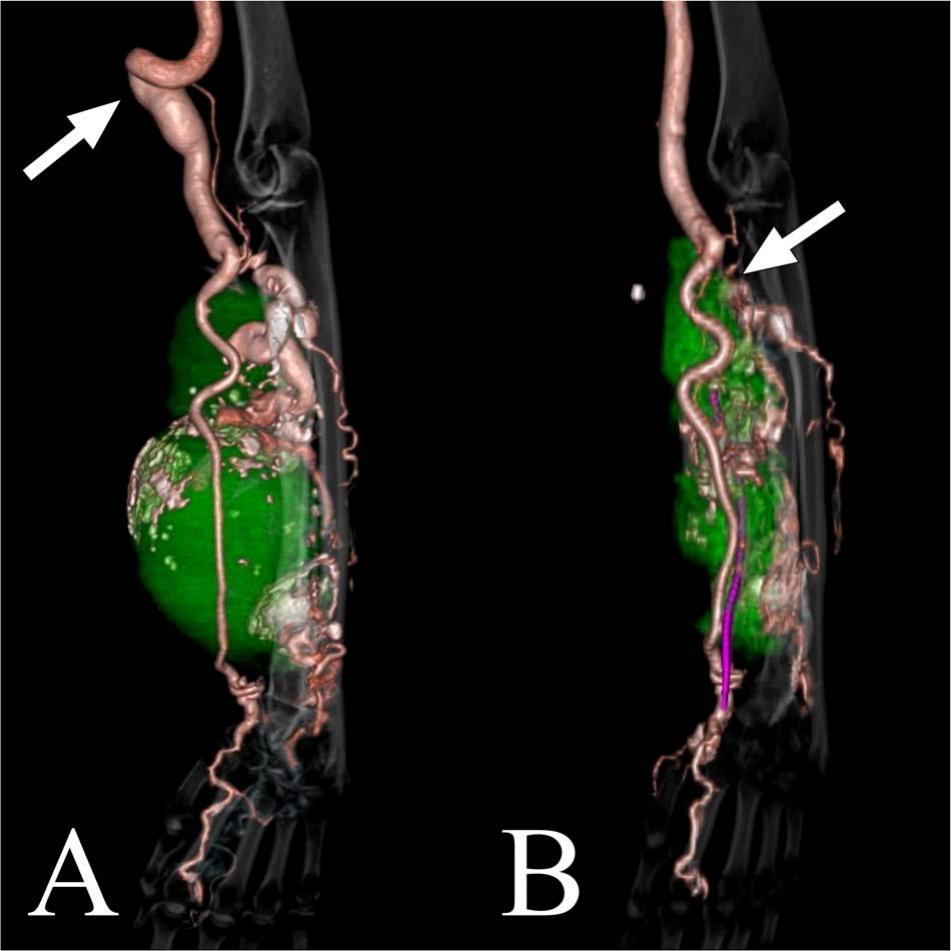
Three-dimensional CT angiography before and after definitive surgical intervention. (A) Preoperative imaging. The arrow indicates the markedly tortuous segment of the brachial artery, which served as the origin of collateral circulation to the aneurysm. (B) Postoperative imaging following endoaneurysmorrhaphy. The arrow indicates a residual collateral vessel, suggesting incomplete occlusion of an inflow source. The purple structure represents the drain inserted into the aneurysmal sac.

Due to the onset of pain, ligation of the ulnar artery proximally was performed 3 years prior (Fig. 2); however, the aneurysm continued to enlarge. Contrast-enhanced computed tomography (CT) revealed collateral vessels from the brachial artery to the aneurysm. Over the following 3 years, the aneurysm further enlarged to 68 × 88 mm, and the forearm circumference increased to 31 cm. As the patient requested definitive treatment, surgical intervention was undertaken (Fig. 3A).

The ulnar artery aneurysm had displaced the surrounding forearm tissues, resulting in distortion of normal anatomical structures. Therefore, preoperatively, the courses of the ulnar nerve, median nerve, and radial artery were identified using ultrasound to avoid injury during the incision.

**Figure 4:**
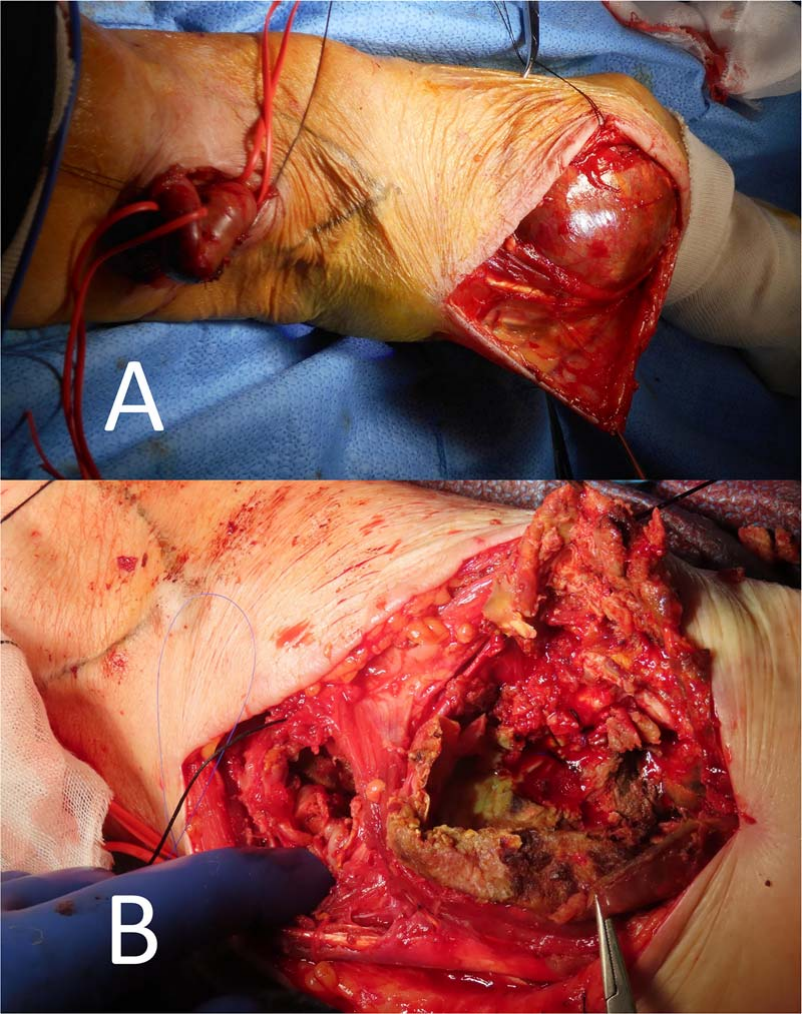
Intraoperative photographs. (A) Overview of the operative field, demonstrating the giant ulnar artery aneurysm and the tortuous brachial artery segment prior to repair. (B) Photograph following incision of the anterior wall of the aneurysmal sac, revealing a large volume of organized thrombus. Muscle tissue is seen traversing the anterior surface of the aneurysmal sac, illustrating the distortion of normal anatomical structures caused by the long-standing aneurysm, which contributed to the technical challenges of achieving complete inflow vessel ligation.

First, the main collateral vessel supplying the aneurysm was controlled at its origin. Because the collateral circulation arose from a markedly tortuous segment of the brachial artery, an incision was made over this segment and the feeder vessel was ligated and divided. The tortuous arterial segment was subsequently resected and reconstructed with end-to-end anastomosis (Fig. 4A).

Next, the anterior wall of the aneurysm was incised, revealing a large volume of organized thrombus. Endoaneurysmorrhaphy was performed, with as much thrombus removed as possible, totaling 235 g. Two inflow arteries within the aneurysm were identified and ligated from inside the sac using polypropylene sutures. A drain was placed within the aneurysmal sac, which was then closed with maximal plication.

Intraoperatively, a sample of the aneurysm wall was submitted for histopathological examination, which revealed transmural sclerosis with an ulcerated atheromatous intimal surface, consistent with an atherosclerotic aneurysm.

The drain was removed on postoperative Day 8, and the patient was discharged home on postoperative Day 10. Mild paresis of the left thumb was observed postoperatively but resolved with rehabilitation. No sensory deficits were noted. Postoperatively, the forearm circumference decreased to 25 cm. Contrast-enhanced CT revealed residual opacification in the central portion of the aneurysm, suggesting the presence of an incompletely ligated inflow vessel (Fig. 3B); therefore, follow-up is being continued.

## Discussion

The present case represents a rare ulnar artery aneurysm associated with congenital AVF and trauma. In vascular access for hemodialysis, dilatation and aneurysmal changes of the inflow artery have been reported, and increased shear stress associated with high-flow conditions has been proposed as a potential underlying mechanism [3]. Trauma may also have served as an additional precipitating factor [4].

The histopathological findings were consistent with an atherosclerotic aneurysm. Although a direct causal relationship between AVF and atherosclerotic changes has not been clearly established, long-standing high-flow conditions associated with AVF may contribute to vascular remodeling and arterial wall degeneration through upregulation of nitric oxide production and outward arterial remodeling [3, 5, 6]. AVF, trauma, and age-related changes likely contributed to aneurysm formation.

The development of collateral vessels supplying the aneurysm, despite proximal ligation, may be attributed in part to the hemodynamic environment created by the long-standing congenital AVF. Experimental studies have demonstrated that AVFs generate elevated fluid shear stress, which has been shown to strongly promote arteriogenesis [7]. This hemodynamic environment likely promoted the growth of collateral vessels, which continued to supply the aneurysm even after proximal ligation.

For the management of this aneurysm, both endovascular and open surgical approaches were considered. Given the giant size of the aneurysm, multiple inflow sources, and severe distortion of surrounding anatomical structures, endovascular exclusion was anticipated to be technically challenging, and surgical volume reduction was considered necessary to achieve symptomatic relief. Open surgical intervention was therefore selected. Among surgical options, complete aneurysm excision was considered impractical because of the marked anatomical distortion, and endoaneurysmorrhaphy was performed, allowing direct identification and ligation of inflow vessels from within the sac while minimizing the risk of injury to adjacent neurovascular structures.

Due to the proximity of the ulnar and median nerves to the aneurysm and the distorted anatomy, preoperative ultrasound mapping of neurovascular structures was employed to minimize the risk of injury [8]. Postoperative neurological deficits were limited to mild thumb paresis, with no associated sensory deficits. The deficit resolved with rehabilitation and was considered most consistent with transient median nerve neurapraxia caused by intraoperative traction or compression, given the close anatomical relationship between the median nerve and the aneurysm. Ultrasound-guided preoperative planning may be particularly valuable in cases where anatomical landmarks are obscured by large or long-standing aneurysms.

Postoperative contrast-enhanced CT revealed residual central opacification, suggesting incomplete occlusion of an inflow vessel. Complete visualization of all inflow vessels was limited by two factors: the need to preserve muscle tissue traversing the anterior surface of the aneurysmal sac (Fig. 4B), and the unacceptable risk of neurovascular injury that more aggressive exploration would have carried given the severely distorted anatomy. This case illustrates the technical complexity of achieving complete aneurysm exclusion in cases characterized by large size, extensive thrombus accumulation, and multiple inflow sources. Additional endovascular intervention may be considered if the aneurysm fails to regress during follow-up.

## Data Availability

Not applicable.
